# Investigating strategies to improve attendance at screening visits in a randomized trial

**DOI:** 10.1186/1745-6215-12-S1-A111

**Published:** 2011-12-13

**Authors:** Fang Chen, K Rahimi, R Haynes, K Naessens, M Taylor-Clarke, C Murray, Jane Armitage

**Affiliations:** 1 Clinical Trial Service Unit & Epidemiological Studies Unit, University of Oxford, OX3 7LF, UK

## Background

A common method of recruiting for randomized trials is to send letters to potentially eligible patients inviting them to a screening appointment. In 3 consecutive UK studies the proportion attending from those invited fell from 49% [1] in 1994-1997 and 42%[2] in 1998-2001 to 13% at the beginning of an ongoing study in 2007. Procedures were similar in the 3 trials except that in 2007 the Patient Information Leaflet (PIL) was enclosed with the invitation letter. In order to understand whether the contents and/or style of invitation would explain the declining trend, 2 separate randomized comparisons were undertaken during the recruitment for the ongoing study.

## Methods

Potentially eligible patients identified from hospital records were randomized to receive either an invitation letter enclosing the PIL (a 12-page A5 MREC approved booklet giving detailed information about the trial) or just a one page summary. A second comparison was made between a PIL modified after Focus Group discussions and the original PIL. Modifications included more colours, pictures and simplified language. The pre-specified endpoints for these assessments were the proportions of patients attending the screening visit, and entering the pre-randomization run-in period.

## Results

Between July and October 2008, 20,759 personalized invitation letters were randomized to have the PIL or brief summary enclosed. There were no significant differences in either the proportions attending: PIL enclosed 1122/10,566 (10.6%) versus not 1181/10,590 (11.2%) [OR 1.06; 95%CI 0.97-1.16]; or in the proportion entering the pre-randomization run-in: 720/1181 (6.8% of those invited) versus 690/1122 (6.5%) [OR 1.05; (0.94-1.17)].

From November 2009 to January 2010, 12,164 patient invitations were randomized to enclose either the modified or the original PIL. A 17% higher attendance was detected for the modified PIL: 580/6104 (9.5%) versus 499/6060 (8.2%) for the original PIL [OR=1.17; (1.03-1.33): p=0.01). However there was no significant difference in the proportion entering the pre-randomization run-in: 373 (6.1% of those invited) vs 339 (5.6%) for modified versus original (OR 1.10; 0.94-1.28).

## Conclusion

Whether the full PIL or brief summary was enclosed with the invitation did not affect the likelihood of attending or entering the run-in. Enclosing a more patient friendly PIL modestly improved the chance of attending, but not whether patients agreed to enter the study.
